# The existence of standard-biased mortality ratios due to death certificate misclassification - a simulation study based on a true story

**DOI:** 10.1186/s12874-016-0112-8

**Published:** 2016-01-22

**Authors:** Andreas Deckert

**Affiliations:** Institute of Public Health, University of Heidelberg, Im Neuenheimer Feld 324, 69120 Heidelberg, Germany

**Keywords:** Bias, Cardiovascular disease, Misclassification, Mortality, Standardized mortality ratio

## Abstract

**Background:**

Mortality statistics are used to compare health status of populations; optimally, they base on individual death certificates. However, determining cause of death is error-prone. E.g. cardiovascular disease (CVD) death determination is characterized by sensitivity (SE) and specificity (SP) lower than 85 %. Furthermore, differential misclassification may be present in case of homogenous target populations. We investigate the bias of standardized mortality ratios (SMR), based on real-world data.

**Methods:**

CVD mortality of 6378 ethnic German repatriates was assessed and the SMR calculated. Non-differential age-dependent misclassification was introduced into data by scenarios of equal SE and SP in a range of 0.7 to 0.85. The bias between originally reported and actual SMR was calculated for each pair of values. Additionally, four differential misclassification scenarios were simulated, reflecting two extreme scenarios of both quality criteria varied in the cohort but fixed to either higher or lower in the reference, and two scenarios of crossed criteria values.

**Results:**

In case of non-differential misclassification the bias is always towards the null-hypothesis. The lowest bias was 13.5 % (SE, SP = 0.85 constantly), the maximum bias was 40 % (SP = 0.7). However, in case of differential misclassification the observed SMR can be on the wrong track. If SP is high but SE low in the cohort, negative bias up to −10 % can occur. In case SE is low but SP is high in the reference, the bias remains always positive. In the opposite case plus SP is high in the cohort, the bias can reach −30 %.

**Conclusion:**

SMR values are always biased due to the diagnostic test character of death determination. In majority of epidemiological studies the bias should be towards the null-hypothesis (non-differential misclassification). However, caution is needed in case of differential misclassification, possibly experienced in studies on homogenous subgroups, and in large prospective cohorts with specifically trained personnel.

## Background

Mortality statistics are a widespread tool to compare and monitor health systems and health status of countries, and are used as a primary alert system to trigger health interventions. Within countries, death statistics are suitable as a first step to identify disadvantaged population subgroups. A recent publication series highlighted the need of high quality civil registration and vital statistics to improve health outcomes [1, 2].

Mortality figures are utilized to derive cause-specific death rates, years of life lost, and healthcare expenditures. These are highly aggregated data, stemming from semi-standardized death certificates, originally developed to fulfil juridical and demographic functions [3]. Initially, a physician who pronounces an individual’s death fills out this predefined form, covering the disease history and diagnosis. The listed diseases are designated as direct or indirect causes, including the underlying disease which caused all the other ailments within a logical chain. However, only the latter is considered as the cause of death in mono-causal statistics, which is still the dominant type of mortality statistics in most countries; additional information available on the death certificate about concomitant diseases is usually excluded from further data processing.

Due to the high level of completeness, death certificates can be used for epidemiological studies. However, data collection and processing differs considerably between countries, and data completeness can only be assumed in high-income countries [4]. Under-registration may be as high as 20 % in some countries in the world, as WHO states in their technical notes. Other sources report about only around 40 % of deaths registered globally [5]. These problems are compounded by unreliable demographic estimates, which affect mortality statistics. Hence, the WHO reminds their data users that “international comparisons between countries and their interpretation should thus be made with caution”.

Although the cause of death determination procedure is internationally standardized to some extent and the subsequent data processing is technically optimized, the process is known to be error-prone for many reasons. For example, cause of death determination and coding the causes of deaths. Death certificate data quality completely depends on the responsible physician filling out the form, and thus on training level and experience in determining the underlying disease [6]. Differences are seen for example if a highly specialized surgeon’s assessment is compared against a general practitioner or an emergency doctor pressed by time [7]. If the distribution of medical specialization is regionally unbalanced (e.g. urban areas with specialized clinics vs. rural areas) then small-scale regional comparisons of causes of death could be biased. Furthermore, physicians may define different underlying causes given the same case, even if they are specialised in the same discipline. Other reasons for bias inherent in death determination are incompleteness or unavailability of patient records for disease history, or lack of autopsies. This is especially true for elderly people, for whom typical age-related causes are often incorrectly assumed to be responsible for the death without taking other reasons into account. Furthermore, the increasing number of multi-morbid patients, which makes it difficult to determine a principal cause of death, complicates death determination.

In the data processing subsequent to cause of death determination, information is coded according to the international statistical classification of diseases and related health problems (ICD). However, although the coding has to be done according to guidelines, it is not immune to errors. One of the main problems that certified ICD coders face is illegible handwriting or the incorrect specification of the causal chain [6]. Other coding errors occur due to coding rules violations, indexing errors, information gaps in external causes, and incorrect determination of the underlying disease [8]. Additionally, even though personnel are technically trained, the results are often heterogeneous and subjective influences cannot totally be excluded. Some studies have been carried out to assess the quality of coding; they report a low inter-observer agreement [9, 10]. Altogether, misclassifications are less frequent on superior ICD coding levels which aggregate the subordinated more specific groups. Misclassifications that occur in each single coding office may appear as random noise or mutually compensate when aggregating data on the national level.

In contrast, the ascertainment errors made by the physicians are primary cause-specific and initial in the cascade, hence will not cancel each other out on aggregated level, but may affect comparative mortality measures of population subgroups within a country. Each single death ascertainment can be regarded as a diagnostic test, with a particular sensitivity (SE) – the proportion of correctly classified deaths among those assigned to that cause, such as e.g. to CVD; and specificity (SP) – the proportion of correctly excluded CVD deaths among all non-CVD deaths. The SE and positive predictive value of cause of death assignments with respect to CVD and ischemic heart diseases (IHD) is known to be worse than for neoplasms. Lloyd-Jones et al. subsequently assessed all fatalities in the well-known Framingham Heart Study using a panel of physicians as reference [11]. The death certificates had a SE of 83.8 % to correctly ascertain coronary heart diseases (CHD) as cause of death and a SP of 84.1 %. However, the physicians in the Framingham community might fill out death certificates more accurately than the average physician due to study ties, rendering a generalisation of these figures too optimistic. Causes of death that the panel of physicians assigned to be indeterminable had often been designated as CHD in the death certificates. The effect was more pronounced among women and the elderly. Among the latter other causes were often hastily excluded. Although the study was run in the US, its results are transferable to other western countries. If one were to assume the proportion of CVD deaths to be 50 % of all deaths in an exposed group and 40 % in an unexposed group (both of same size and same number of overall deaths), a SE of 83.8 % and a SP of 84.1 % for the corresponding death ascertainment would lead to an observed relative risk of 1.16, compared to a true risk of 1.25. Whatever the direction of the true effect is, the resulting bias is towards the null-hypothesis, and more so the rarer the disease, assuming the same SE and SP among both the exposed and unexposed (non-differential information bias).

Furthermore, a population-based autopsy study (autopsy selection bias not present) with 1,060 deceased subjects conducted in East Germany in 1987 also determined an over-representation of deaths due to CVD on death certificates, with the proportion of disagreement higher among the elderly but independent of sex. The SE for the overall CVD group was 83 %, the positive predictive value 69 %, and the three-digit level SE for CVDs only 54 % [12].

Although the existence of bias due to death certificate misclassification is known and studied for relative risks, no investigations of its effect on standardized mortality measures such as standardized mortality ratios (SMR) can be found in literature. Furthermore, it is not clear, whether differential misclassification in the study groups such as migrants and the reference population could lead to completely wrong results.

This simulation study investigates the extent of bias due to non-differential and differential death certificate misclassification. The simulation is based on CVD mortality data which were assessed in 2010 in a retrospective cohort of German repatriates who immigrated to the Augsburg region in Germany between 1990 and 1999. The results have been published elsewhere [13]. The SMR for male German repatriates was 0.82 (95 % confidence interval (CI) [0.65; 1.03]), though this was not significant. Other studies reported similar but significant effects [14–16]. However, a healthy migrant effect most probably was not present [17]. Besides the hardly verifiable assumption of a lifestyle completely different to the autochthonous population in their countries of origin, statistical artefacts could play a role. In order to investigate such a possible bias simulation studies were performed. Misclassification was introduced to the data to calculate the percentage of bias for different scenarios. In the first simulation homogenous misclassification was generated in the migrant group and the reference population (non-differential misclassification). In the second simulation study differential misclassification was applied to the data and the reference population.

## Methods

The study complies with the Declaration of Helsinki. It uses anonymized and aggregated data of a cohort study, for which the study protocol was approved by the ethical committee of Heidelberg University.

CVD mortality (ICD-10 I00-I99) of the German repatriates was retrospectively assessed. Altogether, 6378 ethnic German repatriates were identified in the Augsburg residence registry, immigrating to Augsburg region from the Former Soviet Union between 1990 and 1999. Follow-up and address tracing was done until 10th of May 2010 and the vital status of the individuals ascertained. In case of death, an anonymous copy of the death certificate was requested from local health authorities. Coding of causes of death was conducted by independent coding personnel at the Saarland cancer registry. Reference annual mortality figures of the general German population were obtained from the German Federal Health Monitoring Information System, available in five-year age-groups. Each individual’s time at risk was captured and transferred into accumulated person years (PY) in age and calendar year strata, using a SAS macro [18]. Then the standardized mortality ratios were calculated. The sequencing analyses were restricted to male German repatriates.

In a theoretical situation of age-independent SE and SP the unbiased CVD deaths in a cohort can be calculated according to the following formula, derived from the 2 by 2 table of a diagnostic test:

1 $$ CV{D}_{unbiased}=\frac{{\displaystyle \sum_{i=1}^N{\displaystyle \sum_{j=1}^MCV{D}_{i,j}+SP\cdot D-D}}}{SE+SP-1} $$

Where i refers to the age and j to the calendar year strata, and D are the overall deaths. The same formula can be applied to calculate the true expected CVD deaths based on the expected CVD deaths in the strata. Hence, the true unbiased SMR is as follows:

2 $$ SM{R}_{true}=\frac{CV{D}_{obs, unbiased}}{CV{D}_{\exp, unbiased}} $$

In the specific case of non-differential misclassification and age-independent SE and SP the equation for the unbiased SMR simplifies to:

3 $$ SM{R}_{true}=\frac{{\displaystyle \sum_i^N{\displaystyle \sum_j^M{\underset{obs}{CVD}}_{i,j}}}+SP\cdot D-D}{{\displaystyle \sum_i^N{\displaystyle \sum_j^M{\underset{ \exp }{CVD}}_{i,j}+SP\cdot D-D}}} $$

In reality and in particular in case of CVD deaths the SE and SP are not constant over age but may follow a continuously falling function. Therefore, we conducted simulation studies investigating the effect of age-dependent SE and SP on CVD SMR.

The first part of the simulation study dealt with non-differential death certificate misclassification, as described in scenario A) in Table 1. To simplify matters, the age-dependent misclassification was transferred to a binary scenario, with 70 years of age as the cut-off. The SE and SP were both fixed to 0.85 for CVD deaths occurring before the age of 70 in the cohort and reference population. After the age of 70 years both quality criteria were varied from 0.7 to 0.85, but were of the same magnitude in the cohort and the reference population. The number of the unbiased CVD deaths in each age- and calendar year-stratum of the cohort and in the reference population was estimated according the following equation:

**Table 1:** Non-differential and differential death certificate misclassification simulation scenarios, applied to mortality data of a cohort of migrants in Augsburg, Germany

		Age <70 years	Age ≥70 years	
Misclassification scenario		Cohort & reference	Cohort	Reference
		SE; SP	SE; SP	SE; SP
Non -differential	A	0.85; 0.85	[0.7, 0.85]; [0.7, 0.85]	
Differential	B1	0.85; 0.85	[0.7, 0.85]; [0.7, 0.85]	0.85; 0.85
	B2	0.85; 0.85	[0.7, 0.85]; [0.7, 0.85]	0.7; 0.7
	B3	0.85; 0.85	[0.7, 0.85]; [0.7, 0.85]	0.7; 0.85
	B4	0.85; 0.85	[0.7, 0.85]; [0.7, 0.85]	0.85; 0.7

*SE* sensitivity, *SP* specificity

4 $$ CV{D}_{age, cal}=\frac{CV{D}_{obs}+S{P}_{age}\cdot {D}_{age}-{D}_{age}}{S{E}_{age}+S{P}_{age}-1} $$

Where CVD_age,cal_ is the unbiased amount of CVD deaths in an age- and calendar year-stratum, CVD_obs_ are the observed number of CVD deaths within a stratum, SE_age_ is the age-specific sensitivity, SP_age_ is the specificity, and D_age_ is the amount of overall deaths in the cohort, respectively the reference population, within a stratum. The number of unbiased expected CVD deaths was calculated, as expected according to the null-hypothesis, based on the number of unbiased CVD deaths in the reference population divided by the midyear population of the reference population and multiplied by the PY in the strata of the cohort. The SMR was calculated as the ratio of the observed CVDs and the expected CVDs. The percentage deviation from the originally reported SMR was the bias in each scenario. The results were plotted in three-dimensional grid diagrams.

The second part of the simulation study dealt with the differential death certificate misclassification. The age-dependency of the misclassification was again simplified. However, the SE and SP after 70 years of age in the reference population were kept constant (although on different scales in different simulations) while both measures were varied for the cohort. The subsequent procedure was as above. Here, four extreme scenarios were investigated regarding the reference population (see Table 1): B1) Both SE and SP were 0,85 after 70 years, B2) both quality criteria were 0.7 after 70 years, B3) the SE was 0.7 but the SP was 0.85, and B4) the SE was 0.85 in the reference population after 70 years, but the SP was 0.7.

## Results

The men accumulated around 44,000 person years. Out of 251 deaths, 80 were ascertained to CVD. Lost to follow-up was low at 2.1 %, thus unlikely to bias the results. Altogether 10 death certificates of men were not provided by the local health authorities, introducing some uncertainty. However, correction methods have been applied in the results report [13]. The simulation results are not at all affected by these missing values.

### Non-differential misclassification

**Fig. 1:**
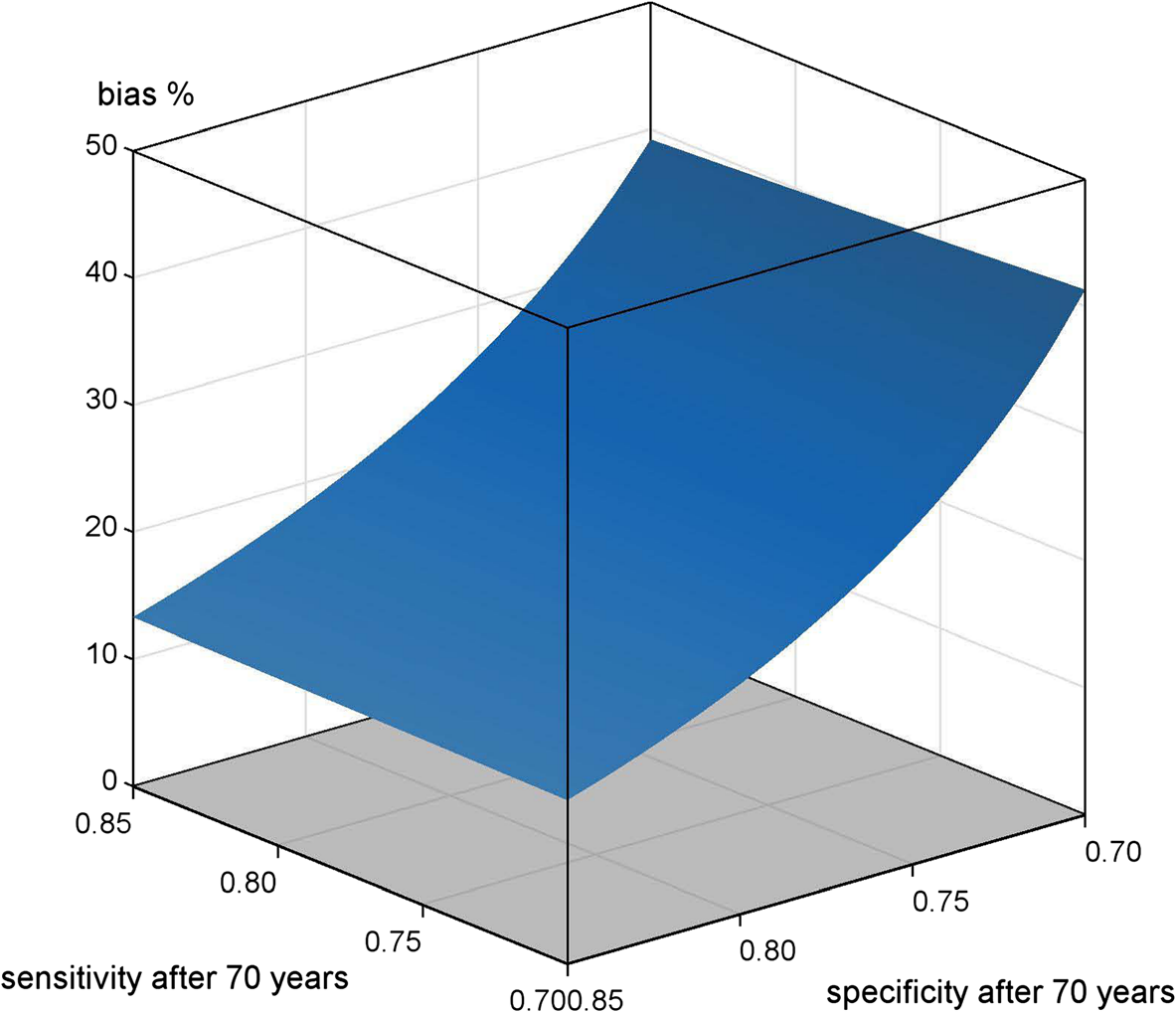
Scenario A) non-differential misclassification; < 70 years SE, SP = 0.85; ≥70 years SE = [0.7, 0.85], SP = [0.7, 0.85]; applied to mortality data of a cohort of migrants in Augsburg, Germany

Figure 1 depicts the results of scenario A), see Table 1.

If no age dependency is present i.e. SE, SP are kept constant at 0.85, the bias is around 13.5 %. Besides the specific case of non-differential misclassification and age-independent SE and SP, which corresponds to the left corner of the surface in Fig. 1 and relates to equation 3) where SE has no influence, the influence of age-dependent SE is always negligible in comparison to SP. If SP is low at 0.7, the bias is around 40 %. However, the bias is always of positive value, meaning the actually observed SMRs are biased towards the null hypothesis.

### Differential misclassification

**Fig. 2:**
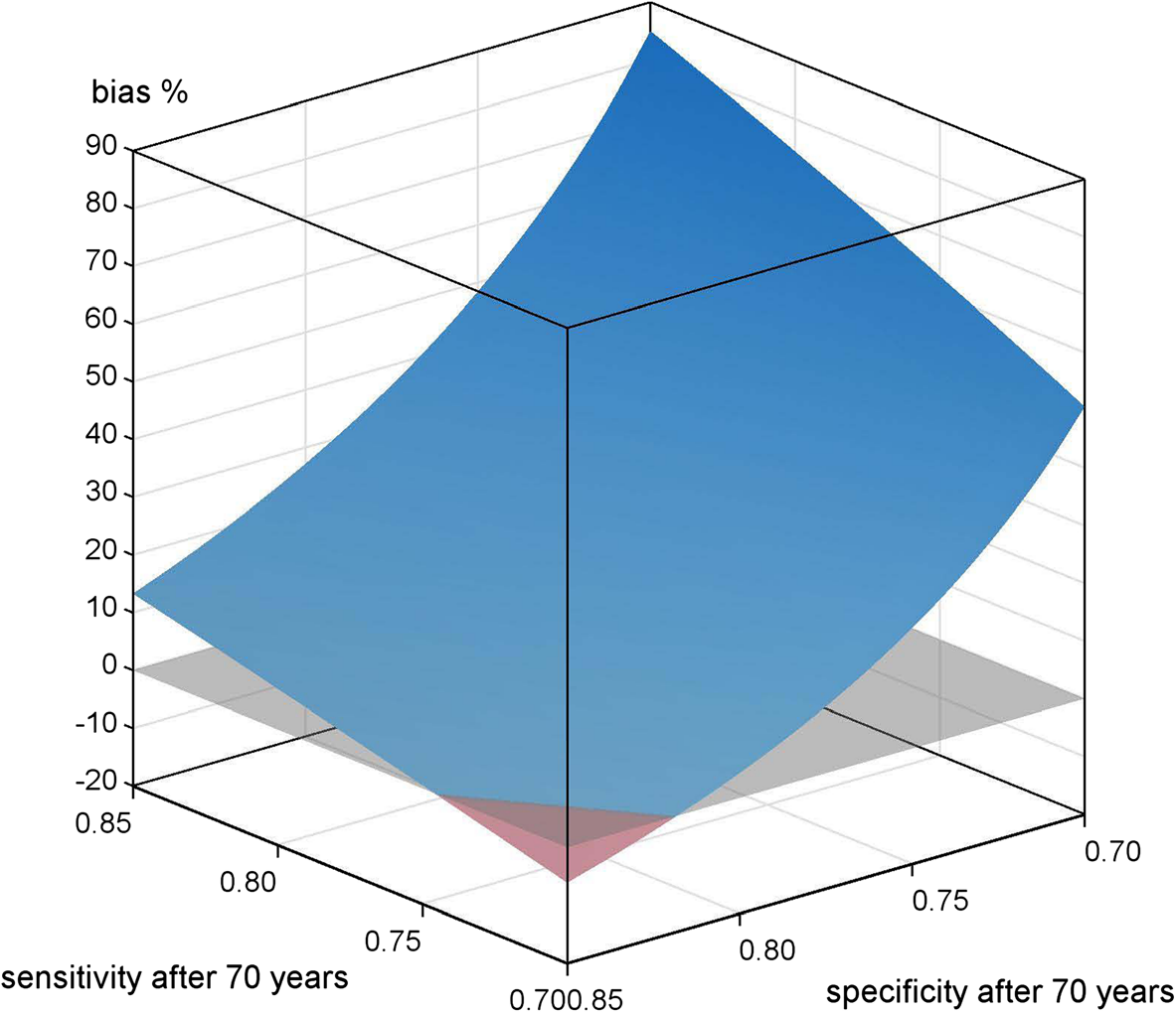
Scenario B1) differential misclassification; <70 years SE, SP = 0.85; ≥70 years cohort SE = [0.7, 0.85], SP = [0.7, 0.85], reference SE, SP = 0.85; applied to mortality data of a cohort of migrants in Augsburg, Germany

Figure 2 contains the simulation results of scenario B1), see Table 1.

The bias has positive values for the majority of the quality criteria pairs. If SP is kept constant but SE is varied, the bias decreases with lower SE. Additionally, a small proportion of specific combinations can actually cause negative bias down to approximately −5 %. This is true for low SE (smaller 0.74) in combination with a higher SP (larger 0.82) in the cohort.

**Fig. 3:**
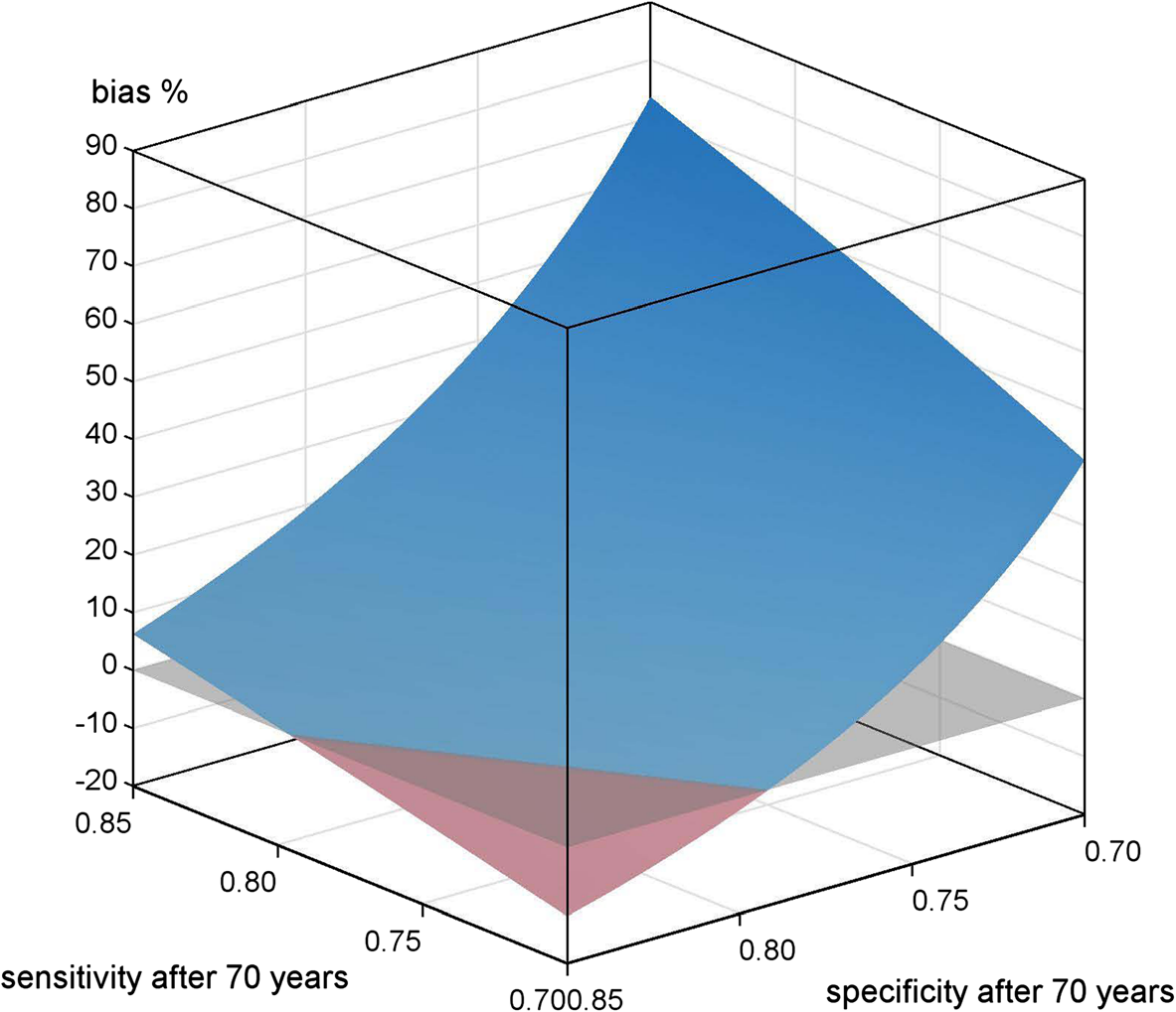
Scenario B2) differential misclassification; <70 years SE, SP = 0.85; ≥70 years cohort SE = [0.7, 0.85], SP = [0.7, 0.85], reference SE, SP = 0.7; applied to mortality data of a cohort of migrants in Augsburg, Germany

Figure 3 depicts the simulation results of scenario B2), see Table 1.

The plot surface follows the distribution observed in Fig. 2 but shifted downwards. The SE borderline to negative bias is 0.79 here and the corresponding SP is 0.79.

**Fig. 4:**
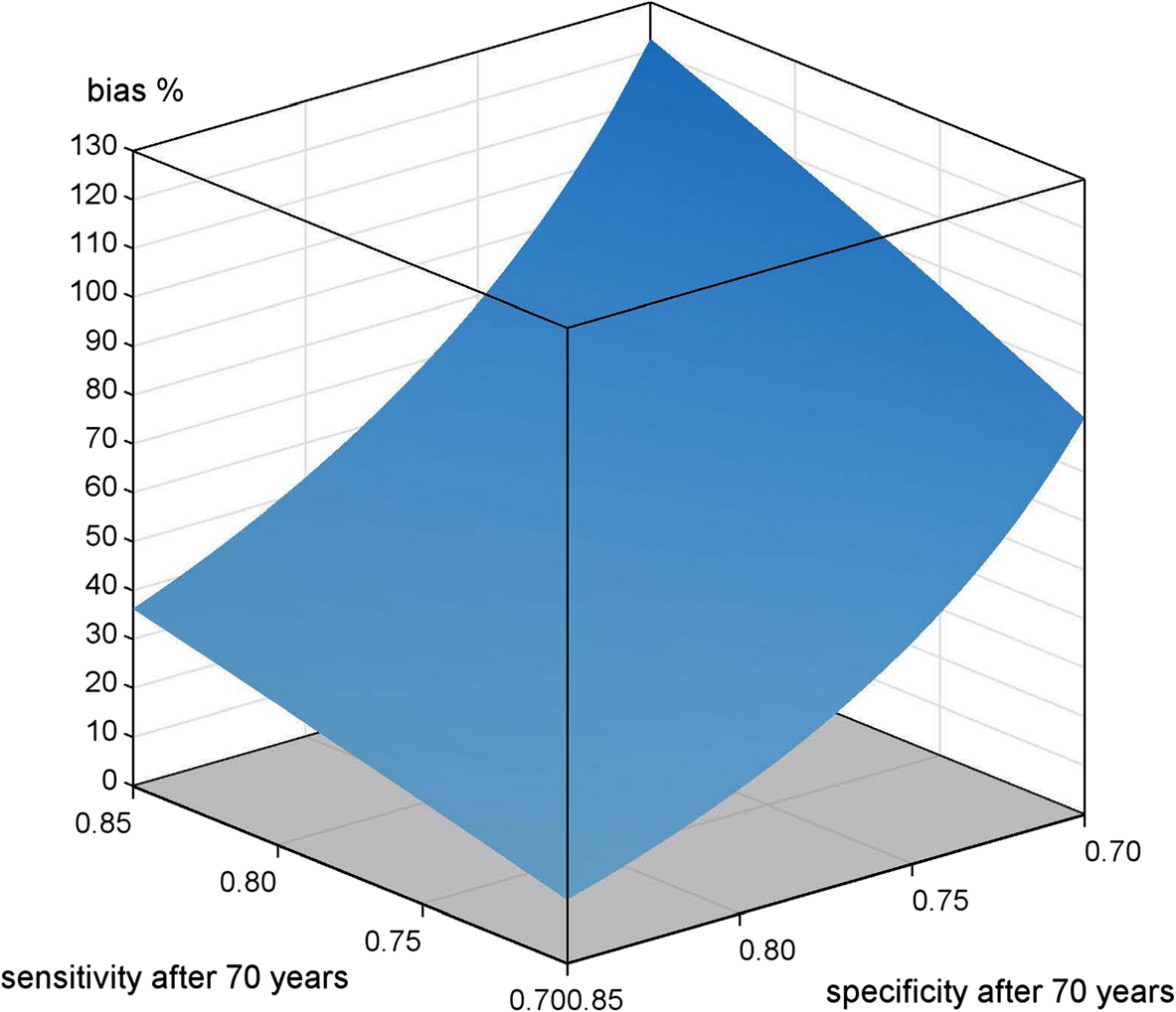
Scenario B3) differential misclassification; <70 years SE, SP = 0.85; ≥70 years cohort SE = [0.7, 0.85], SP = [0.7, 0.85], reference SE = 0.7, SP = 0.85; applied to mortality data of a cohort of migrants in Augsburg, Germany

Figure 4 depicts the simulation results of scenario B3), see Table 1.

**Fig. 5:**
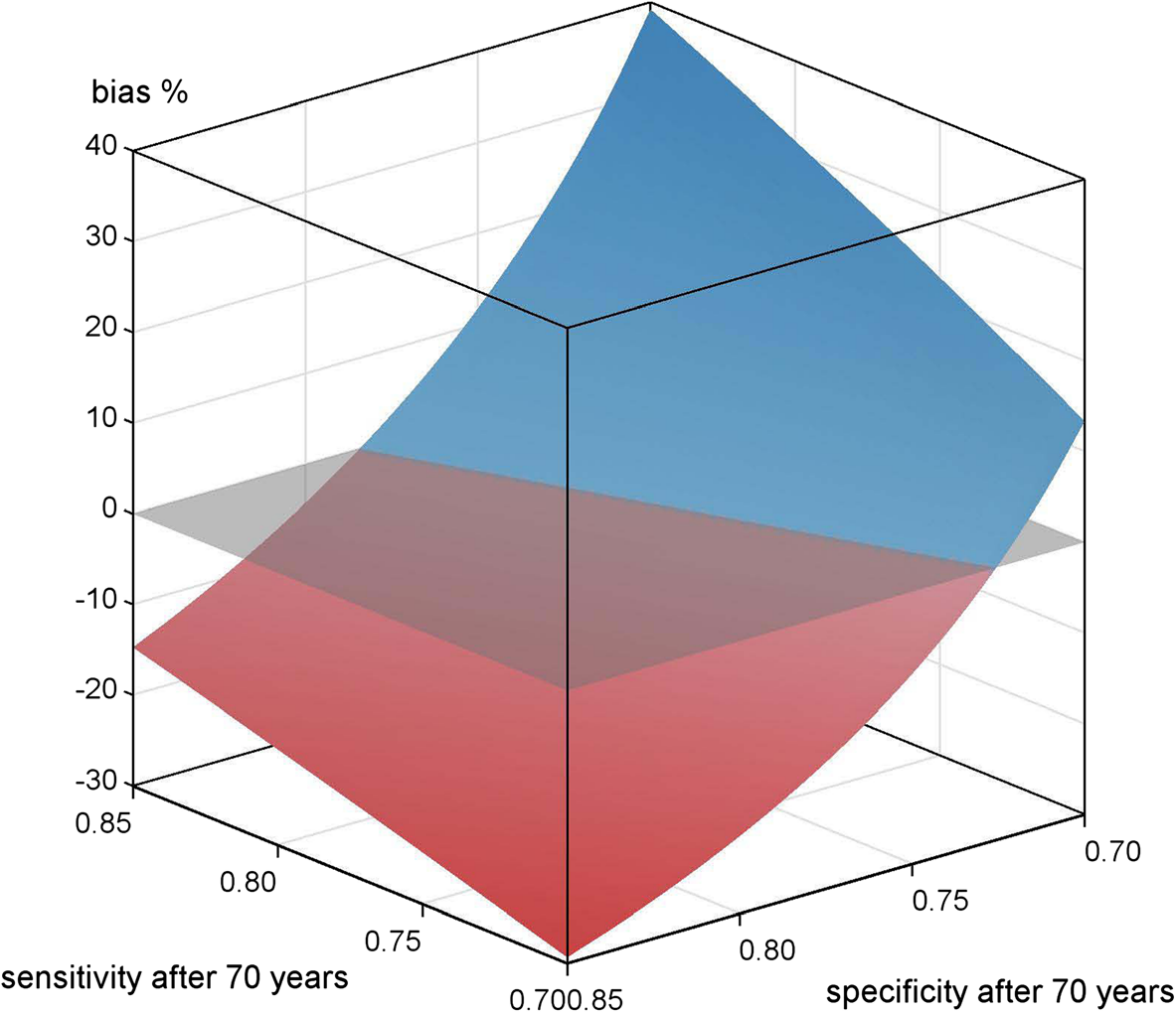
Scenario B4) differential misclassification; <70 years SE, SP = 0.85; ≥70 years cohort SE = [0.7, 0.85], SP = [0.7, 0.85], reference SE = 0.85, SP = 0.7; applied to mortality data of a cohort of migrants in Augsburg, Germany

The graph appears almost similar to the Figs. 2 and 3 but shifted upwards now; there is no negative bias visible at all.

The last graph (Fig. 5) shows the results of scenario B4), see Table 1.

The situation of reversed SE and SP in the reference population completely changed the picture. Now the majority of combination pairs result in negative bias. In particular, pairs of higher SE and SP in the cohort set the actually observed SMR on the wrong track. Only if SE is high enough, a low SP keeps the bias positive. If SE is 0.7, then the negative bias can reach −30 %.

## Discussion

This study addresses the often neglected issue of hidden bias inherent in SMR calculations due to the diagnostic test character of the utilized death certificates. If the process of death certificate misclassification is similar in the cohort and the reference population, the bias is always towards the null hypothesis. Hence, the actually observed SMR underestimates the true effect, but depending on the level of SE and SP, the underestimation can be considerably high. However, if death certificate misclassification is different in the cohort and the reference population, the actually observed SMR can even lead to completely wrong conclusions.

Applied to the published results of the study, the true but unknown SMRs are different to those reported (see Table 2).

**Table 2:** Effect of SE, SP misclassification on the estimated SMR of 0.82, 95 % confidence interval [0.65; 1.03] in a cohort of migrants in Augsburg, Germany

	Age ≥70 years		
	Cohort	Reference	True SMR [95 % CI]
	SE; SP	SE; SP	
Non -differential	0.7; 0.7	0.7; 0.7	0.58 [0.42; 0.77]^a^
Differential	0.7; 0.7	0.85; 0.85	0.54 [0.37; 0.73]^a^
	0.85; 0.85	0.7; 0.7	0.76 [0.59; 0.99]^a^
	0.85; 0.7	0.7; 0.85	0.37 [0.26; 0.51]^a^
	0.7; 0.85	0.85; 0.7	1.16 [0.91; 1.46]

*SE* sensitivity, *SP* specificity

^a^significant result

If one assumes that strong non-differential misclassification was present after 70 years, the true and significant SMR would be 0.58, in contrast to the actually observed non-significant SMR of 0.82. Assuming both SE and SP after 70 years were low in the cohort but high in the reference population, the true but unknown SMR would be 0.54. In the opposite case of both quality criteria were low in the reference population but high in the cohort, the true SMR would be 0.76. If SE is 0.85 and SP is 0.7 in the cohort and vice versa in the reference population, then the true SMR would be 0.37. Finally, if SE is 0.7 but SP 0.85 in the cohort and vice versa in the reference population, then the true SMR would be 1.16 (CI: 0.91, 1.46), thus opposite to the observed effect. Hence, in the majority of the presented extreme scenarios the results reported would be confirmed and strengthened. However, in the latter scenario the interpretation of the true results albeit not significant would be completely opposite than actually observed. Nevertheless, the German repatriate studies mostly reported significantly lower overall mortality, and CVDs accounted for the majority of deaths. Hence, a lower CVD mortality accompanied by a lower overall mortality most likely shows the true picture.

Of course age-standardization takes care of bias caused by age but not by age-dependent misclassificiation. This is because misclassification is inherent within each age group. Hence, the low SMR estimate observed in the male German repatriate cohort is not caused by potentially less wrongly certified CVD deaths due to a lower mean age at death, as this was compensated for by age-standardization. But if the prevalence of lethal CVD in the cohort differed from that in the general German population due to risk factors other than age, the misclassification led to biased rate ratios in all age groups and to a biased overall SMR.

Death certificate misclassification is present in each trial presenting death statistics. However, in vast majority of epidemiological studies which investigate the mortality pattern of population subgroups there is sound reason to assume that the presence of misclassification is of similar magnitude in both the cohort and the reference population, resulting in underestimated effects. For CVD deaths the proportion of false positives in (retrospective) cohorts and in the reference population can be quite high, due to common misclassification in the elderly, for instance. Since the observed biased CVDs usually account for a large proportion of the overall deaths, this results in a low CVD SP of the death certificates. And since a high level of SE cannot compensate the bias caused by a low SP, in particular CVD death statistics might be affected by constantly underestimated effects.

In some specific settings differential misclassification might actually be present. This may be true if e.g. a closed subgroup of a population is investigated, such as some densely settling migrants with a high proportion of peer physicians. German repatriates for example are known to frequently settle in high peer density areas, with several physicians belonging to the same group. Repatriates often prefer to be treated by these physicians only (qualitative observations in the Augsburg cohort). Thus, a high proportion of deaths in the cohort might have been ascertained by these physicians, though less true for deaths caused by severe diseases, occurring in hospitals. Usually, immigrating physicians have to apply for acceptance of their profession at the so-called *Landesärztekammer* in Germany, and must prove their knowledge after a practical year and an examination [19]. However, for German repatriates special regulations applied. According to the differences in medical training between their country of origin in the Former Soviet Union and Germany, the repatriate physicians might have ascertained causes of death generally somewhat differently. In consequence, the SE and SP of death ascertainment in the cohort could actually differ to those valid in the general population, leading to differential misclassification. Another study used indirect indicators to show that death recording within the German repatriates might be different [20]. Hence, it cannot completely be ruled out that the applied criteria for death ascertainment differ. However, if misclassification is worse within the cohort, the bias still tends to be positive, with exceptions for scarce extreme scenarios. In contrast, the virtually constructed scenario of a cohort with low SE but high SP combined with the opposite conditions in the reference population where the negative bias prevails seems to be improbable but still possible in certain situations. However, usually in rare scenarios where a negative bias is present, the negative bias close to zero is often accompanied by a non-significant confidence interval, which additionally can protect from drawing wrong conclusions.

Although the majority of the results presented here relieve epidemiological studies from the risk of reporting wrong results due to negative bias, SMR analyses in prospective cohorts should be exercised with caution. In such settings the SE and SP in the cohort might perform better than in the reference population due to study related learning effects. Although the area of critical differences to the quality criteria in the reference population seems to feature improbable scenarios, the actual assessment of the quality criteria in a pilot study within big cohorts is clearly advisable.

The simulation scenarios were limited to four extreme situations present in the reference population. However, since the results of other scenarios than these should lie in between, these extreme scenarios seem to be sufficient to mark out the areas of dangerous situations. In case the quality criteria combinations are even further located below or above the limits of 0.7 and 0.85, the values attributed to the bias are in the prolongation of the plot surfaces.

The effect of death certificate misclassification on observed SMRs and thus on the magnitude of bias depends on the existing SE and SP values but not on the sample size. The latter influences only the width of the CIs of SMR and bias which will differ from study to study. Consequently, presenting the study specific CI surfaces in the graph would not have added a general value to the results. Hence, bias CIs were omitted in the graphs, and thus areas of final rejection of the null-hypothesis are not visible.

This simulation study has a strong link to real world study settings as it bases on actual observed data. The magnitude of misclassification introduced is around the estimates observed in representative studies such as the Framingham Heart Study. The bias estimates derived from the two-step age-dependent SE and SP approach are closer to reality than bias calculations based on the assumption of a constant SE and SP over age, in particular in case of CVD deaths. However, in reality the gradients might follow continuously falling functions, hence, the two-step approach bias probably still overestimates the effect in some situations. Nevertheless, the results are of highly practical interest and should be considered when discussing possible bias in mortality statistics. In particular, large prospective cohorts should investigate death certificate misclassification in sub-samples and implement measures to avoid differential death certificate misclassification.

## Conclusion

Death certificate misclassification is known to bias mortality figures. E.g. sensitivity and specificity of cardiovascular diseases death determination is low around 85 %. This study investigates the effects of non-differential and differential misclassification on SMR, based on simulation scenarios. In majority of studies with non-differential misclassification present, bias in SMR is towards the null-hypothesis. Particular attention should be paid by the health system to reduce the number of false positive CVD deaths, thus to increase CVD SP values in general. However, differential misclassification, which is possible in large prospective cohorts with learning curve effects, requires caution. In specific settings the SMR estimate could show an effect opposite to reality.
